# BK polyomavirus infection: more than 50 years and still a threat to kidney transplant recipients

**DOI:** 10.3389/frtra.2024.1309927

**Published:** 2024-01-24

**Authors:** Sandesh Parajuli, Fahad Aziz, Weixiong Zhong, Arjang Djamali

**Affiliations:** ^1^ Division of Nephrology, Department of Medicine, University of Wisconsin School of Medicine and Public Health, Madison, WI, United States; ^2^ Department of Pathology and Laboratory Medicine, University of Wisconsin School of Medicine and Public Health, Madison, WI, United States; ^3^ Department of Medicine, Maine Medical Center Maine Health, Portland, ME, United States

**Keywords:** acute kidney injury (AKI), antibody-mediated rejection (AMR), BK polyomavirus (BKPyV), BKPyV-associated nephropathy (BKPyVAN), BKPyV management

## Abstract

BK polyomavirus (BKPyV) is a ubiquitous human polyomavirus and a major infection after kidney transplantation, primarily due to immunosuppression. BKPyV reactivation can manifest as viruria in 30%–40%, viremia in 10%–20%, and BK polyomavirus-associated nephropathy (BKPyVAN) in 1%–10% of recipients. BKPyVAN is an important cause of kidney graft failure. Although the first case of BKPyV was identified in 1971, progress in its management has been limited. Specifically, there is no safe and effective antiviral agent or vaccine to treat or prevent the infection. Even in the current era, the mainstay approach to BKPyV is a reduction in immunosuppression, which is also limited by safety (risk of *de novo* donor specific antibody and rejection) and efficacy (graft failure). However, recently BKPyV has been getting more attention in the field, and some new treatment strategies including the utilization of viral-specific T-cell therapy are emerging. Given all these challenges, the primary focus of this article is complications associated with BKPyV, as well as strategies to mitigate negative outcomes.

## Introduction

BK polyomavirus (BKPyV) is a ubiquitous human polyomavirus, which is a major viral pathogen after kidney transplantation (1). The first case of BKPyV was diagnosed in an immunosuppressed kidney transplant recipient who presented with ureteric stenosis in 1971 and is named after the initials of this kidney transplant recipient (2). The primary BKPyV infection occurs in early childhood, mainly without any symptoms or only with mild respiratory symptoms and persists in latent form in the kidneys and urogenital tract (3). BKPyV replicates in renal tubular cells, along with other glomerular vascular units including podocytes, endothelial, and mesangial cells (4). After kidney transplantation, the virus becomes reactivated because of immunosuppression and begins to replicate as a result of the breach in the uroepithelium. This sets off a chain reaction of events that begins with tubular cell lysis and viruria, followed by viremia, and BKPyV-associated nephropathy (BKPyVAN) in the absence of intervention (5, 6). After kidney transplantation, BKPyV reactivation could manifest as viruria in 30%–40%, viremia in 10%–20%, and BKPyVAN in 1%–10% of the recipients (7). In addition, it is estimated that 10%–80% of the recipients with BKPyVAN lose their graft prematurely (8). In this article, we will discuss the risk factors and complications associated with BKPyV infection, as well as some of the advances and pitfalls in management that are associated with it.

### BK serology

BK polyomaviruses are a species of icosahedral, non-enveloped, double-stranded DNA viruses. The genomes of all known full-length isolates of BKPyV can be categorized into four discrete genotypes (I–IV) based on analyses of nucleotide sequences (9). The prevalence and sequence characteristics of each genotype are thought to vary within different human populations worldwide (10). Polyomavirus seropositivity is common in the United States and varies by sociodemographic and biological characteristics, including those related to immune function. In one study among 460 participants, 87.6% were seropositive for BKPyV (11). BKPyV-I is the most common genotype and studies indicate 83%–98% of individuals have antibody responses to BKPyV-I major capsid VP1 by the time they are 21 years old (12, 13). However, after kidney transplantation with T-cell suppression, recipients are vulnerable to developing new serotype BKPyV infections, mainly type IV (12). Polymerase chain reaction (PCR)-based prevalence studies have suggested that infection with BKPyV-II or BKPyV-III is rare in all human populations worldwide (14, 15). However, another study based on the serological analysis indicates the prevalence of BKPyV-II seropositivity of 69% and 42% for BKPyV-III (12). BKPyV genotype mismatch between recipients and donors and lower titer of genotype-specific neutralizing antibody titer are two of the predictive markers for BKPyV replication post transplant (16). However, in clinical practice accepting or rejecting organ transplantation based on the BKPyV genotype is not a widespread practice, as it may add more complexity to already limited donor organs.

### Risk factors

Immunosuppression intensity is currently the only widely accepted risk factor for BKPyV replication (17). Another important risk factor is the proximity by time to the transplantation, as the majority of BKPyV replication occurs within the first 1–2 years of transplantation (18). In addition, kidney transplantation itself is one of the most important risk factors for BKPyV replication. Outside of kidney transplantation, BKPyV is mostly encountered in bone marrow transplant recipients, mainly presenting as hemorrhagic cystitis (19). However, there have been few case reports and series on BKPyV viruria and viremia among other solid organ transplant recipients, including the heart, lung, or liver, without significant detrimental kidney function in these patients (5). The existing body of research has compiled a comprehensive list of additional potential risk factors; however, the majority of these risk factors are either ambiguous, inconsistently identified across studies, or contradicted by findings from different studies: for example, tacrolimus-based immunosuppression regimens, deceased donor recipients, male recipients, acute rejection, and ureteral stent placement, donor–recipient human leukocyte antigen (HLA) mismatch >4, donor BKPyV seroreactivity, older recipient, previous transplant, steroid pulses, and many more (20). On the other hand, Drachenberg et al. found an inverse relationship between the level of HLA matches and graft survival among recipients with BKPyVAN. Recipients who maintained graft function had a lower mean HLA match of 1.5 vs. 2.87 among those who lost their graft (*p* = 0.001), thus postulating the lack of HLA matches as a predictor of better outcomes in patients with BKPyVAN (21). Some of the risk factors for post-transplant BKPyV replications are summarized in Table 1 (22, 23).

**Table 1:** Risk factors for BKPyV replication after kidney transplantation.

Immunosuppression related	Types and degree of immunosuppression
	Depleting induction therapy
	Treatment of rejection
Transplant related	Post-transplant interval
	Prolonged ischemia time
	Ureteric stent placement
	Graft injury, re-operation
	Degree of HLA mismatch
	ABO incompatibility
Donor related	Older donor age
	Donor BK seropositivity
	Absence of HLA-C7
	Deceased donor
Recipient related	Older recipient age
	Obesity
	Previsions graft failure due to BKPyVAN
	BK seronegative
	Degree of HLA mismatching
	Pre-transplant serum albumin level
	Negative virtual cross match and lower panel reactive antibodies

Further, evidence suggests BKPyV viremia is predominantly donor-derived rather than a reactivation of the recipient's latent infection (24). In one contemporary study among deceased donor kidney transplant recipients, where both kidneys were transplanted in the same single center, Breyer et al. noticed a higher donor body mass index to be protective against BKPyV viremia, and having concordance or discordance of BKPyV viremia in the recipients receiving deceased donor kidneys from the same donor in two different recipients was not associated with inferior outcomes (20). Similarly, in another study, Srivastava et al. noticed, pre-transplant hypoalbuminemia to be one of the risk factors for post-transplant BKPyV viremia (25). Likewise, in a recent study, even having a kidney-delayed graft function was associated with an increased risk for BKPyV viremia (26). Some studies suggest prolonged cold ischemia time to be a risk factor for BKPyV replication, while others do not (17, 27, 28). In addition, in a pre-clinical animal model, donor acute kidney injury (AKI) was associated with an increased risk for post-transplant BKPyV replication (29). However, contrary to this, in one multicenter study among 1,025 kidney recipients, Hall et al. reported, that donor AKI was associated with a lower risk of BKPyV viremia (30). In light of all these conflicting and puzzling data, the only risk factor that has been shown to consistently be associated with post-transplant BKPyV replication is the degree of immunosuppression in kidney transplant recipients, primarily within the first few months after receiving the transplant.

### Management

#### Diagnosis and screening

In general, BKPyV replication post transplant is asymptomatic and diagnosed with routine screening. Screening for BKPyV replication, whether in urine or plasma, followed by the timely reduction of immunosuppression is the only currently established option to prevent detrimental outcomes from BKPyV infection (31). Most, if not all, of the kidney transplant programs implement regular screening for BKPyV replication. However, there are variations in the frequency, timing, and initial methods used for BKPyV screening. Both the Kidney Disease Improving Global Outcomes and the American Society of Transplantation Infectious Diseases Community of Practice have published screening recommendations, highlighting the importance of the early detection of DNAemia (5, 32). Some of the commonly used screening tests include screening for decoy cells in the urine, quantification of urine BKPyV DNA by real-time PCR, and quantification of plasma BKPyV DNA by PCR (5, 33). All these tests have their pros and cons. Some centers start screening with urine PCR, given the high sensitivity and less invasive nature of this test, and proceed to plasma PCR for those with positive viruria, while other centers start screening with urine decoy cells, due to their high sensitivity and negative predictive value for the diagnosis of BKPyVAN at 100%. However, quantification of plasma BKPyV DNA by real-time PCR is the preferred screening test for BKPyVAN at most transplant centers due to it being both highly sensitive (100%) and specific (88%) for the diagnosis of BKPyVAN along with a higher positive predictive value than viruria or decoy cells (34). In addition, Haller et al. recently reported on C-X-C motif chemokine 10 (CXCL10), which is a small cytokine belonging to the CXC chemokine family, and found a stepwise rise in the median urine CXCL10 levels at various phases of BKPyV replication (35). However, moving forward it will be of interest to assess the importance of the early detection of BKPyV by CXCL10 in overall patient and graft outcomes. As studies suggest, just having lower level BKPyV viremia without BKPyVAN may not have a detrimental effect (36).

For a molecular characterization of BKPyV, it is essential to identify the genotypes of the virus. This will help analyze the distribution of the variants of the virus in each population as well as help determine the mismatches in the genotypes between recipients and donors. A common method of genotyping BKPyV is sequencing, which was first described by Furmaga et al. (37). With the advancement of research and knowledge of the genetic variation in BKPyV, the sequencing reaction is used for further division into subtypes of the virus (38). These recent achievements, including advancements in genomic techniques, have contributed a better understanding of the course of infection and the molecular epidemiology of BKPyV, which will help identify the risk and proper management of this virus.

#### Treatment

**Figure 1:**
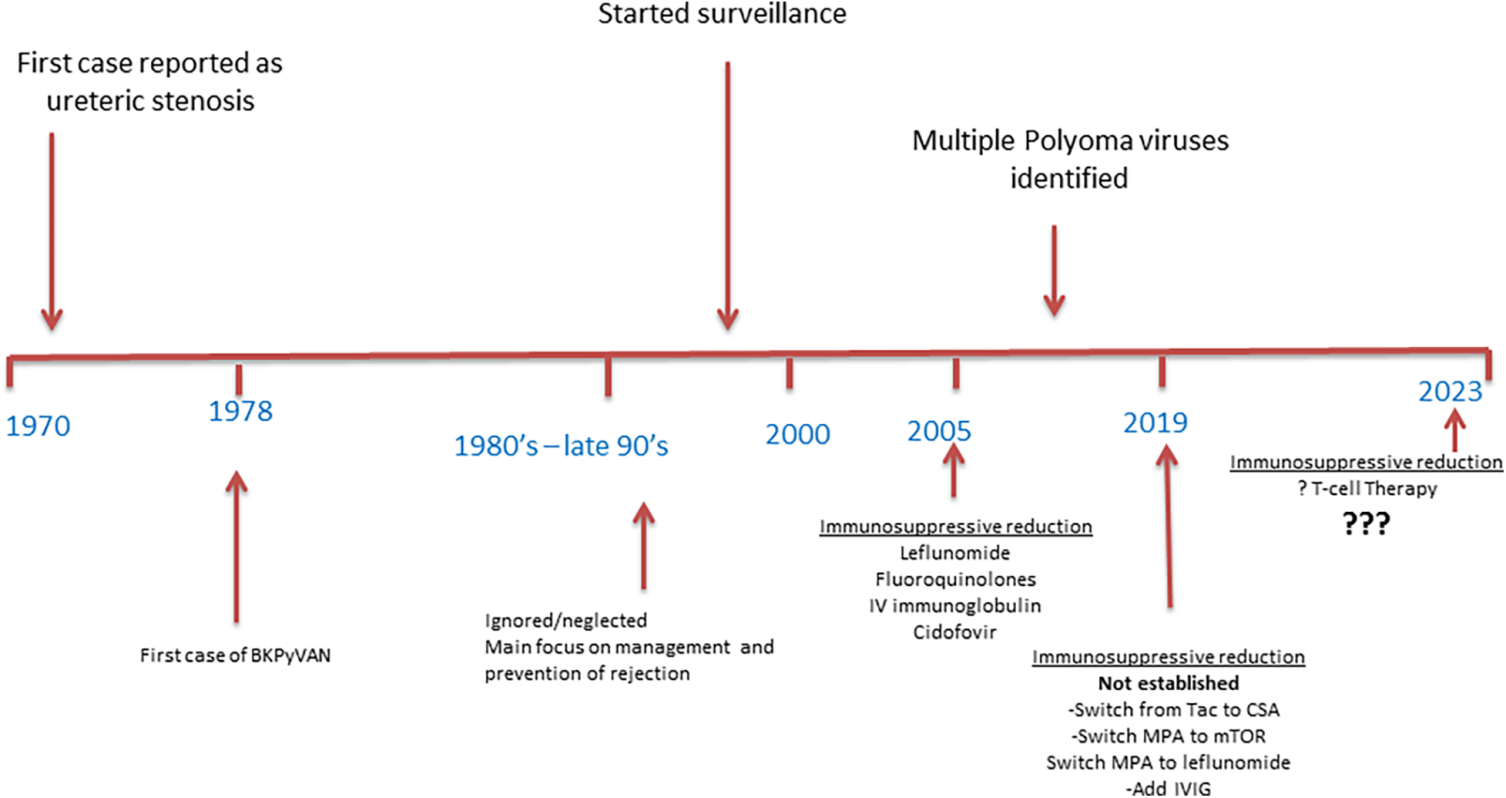
Evolution of BK polyomavirus management.

The timelines and evaluation of various treatments of BKPyV are summarized in Figure 1 (6, 39, 40). Unfortunately, since the first report of BK virus-related complications more than 50 years ago, little progress has been made as no effective medications exist for either treatment or prophylaxis (5). To date, the mainstay of treatment for severe BKPyV or BKPyVAN is the reduction of immunosuppression (41), because adjuvant therapies to treat BKPyV replication have not been safe and effective, with a lack of rigorous studies addressing the role of leflunomide, cidofovir, intravenous immunoglobulin (IVIG), switching from tacrolimus to cyclosporine or mycophenolic acid to mTOR inhibitors, the use of fluoroquinolones, and many more (31, 40). Usually, the antimetabolite is reduced or discontinued, followed by a reduction of calcineurin inhibitors trough goal. The timelines and evaluation of various treatments of BKPyV are summarized in Figure 1 (6, 39, 40).

Even the reduction of immunosuppression may not be safe and effective in all recipients. In one study, among 224 kidney transplant recipients with initial plasma BKPyV-DNAemia >3 log10 copies/ml (>1,000 copies/ml), Kharel et al. reported that even after the initial and stepwise reduction in immunosuppression, only 53% were able to clear viremia without major complications within 2 years post transplant (42). However, of the remaining recipients, 19% either developed *de novo* donor-specific antibodies against the HLA antigen or rejection, indicating an aggressive reduction of immunosuppression, while the remaining 28% developed a severe form of BKPyV with BKPyV-DNAemia >5 log10 copies/ml or even BKPyVAN, indicating an inadequate reduction of immunosuppression. With this, the authors recommended a personalized immunosuppressive modification plan based on patient-specific risk factors to prevent any detrimental outcomes associated with BKPyV (42). In addition, BK virus-specific T-cell therapy (VST) may be a promising addition for the management of post-transplant BKPyV infections in the near future, as it is mainly used only in clinical trials currently (43). The use of adoptive allogeneic T-cell transfer is a therapeutic option capable of restoring virus-specific T-cell immunity with infusions of VST from donor-derived VSTs (44). VSTs have been used and found to be a safe and effective treatment of viral infections in hematopoietic stem-cell transplantation for more than two decades (43, 45). Although it is relatively new among kidney-only transplant recipients with BKPyV, there are few clinical trials underway, including one from the University of Wisconsin (NCT03950414) and another from the University of Cincinnati (NCT02532452) regarding the utilization of VST for the management of severe cases of BKPyV (43). Both centers are actively recruiting patients and outcomes data have not been released yet. We hope to see positive reports of this soon.

### Complications associated with BKPyV replications

Viruria is the earliest manifestation of BKPyV infection, is mostly asymptomatic, and is without any clinical consequence (46). In one study, urine viral loads <7 log10 copies/ml did not progress to high viral loads of BKPyV viremia or BKPyVAN and did not show a signiﬁcantly negative impact on the kidney graft function (47). Though viruria is non-specific, at higher levels it could be a sensitive marker for progression to BKPyVAN (48). Similarly, urine decoy cells, which are renal tubular or uroepithelial cells containing intranuclear viral inclusions, precede BKPyV viremia and BKPyVAN; however, like viruria, the detection of decoy cells is non-specific (49).

BKPyV viremia follows viruria and usually with a high urine viral load. Similar to viruria, viremia is also asymptomatic (50). Viremia is a better predictor of progression to BKPyVAN in comparison to viruria (51). Although BKPyV viremia is asymptomatic, studies report that the management of BKPyV viremia is associated with an increased risk for the development of *de novo* donor-specific antibodies (52, 53). In another study among 1,146 kidney transplant recipients, the authors analyzed the outcomes of death, graft failure, rejection, and other opportunistic infections based on the no detectable viremia vs. various levels of BKPyV viremia and BKPyVAN, and reported that BKPyV viremia without BKPyVAN was not to be associated with an increased risk of outcomes of interest (36). However, in the same study, comparing outcomes comparing BK PCR >10,000 vs. <10,000 copies/ml within the first year of the transplant was associated with an increased risk of other infections, mainly urinary tract infections among higher levels of the BKPyV group (36).

**Figure 2:**
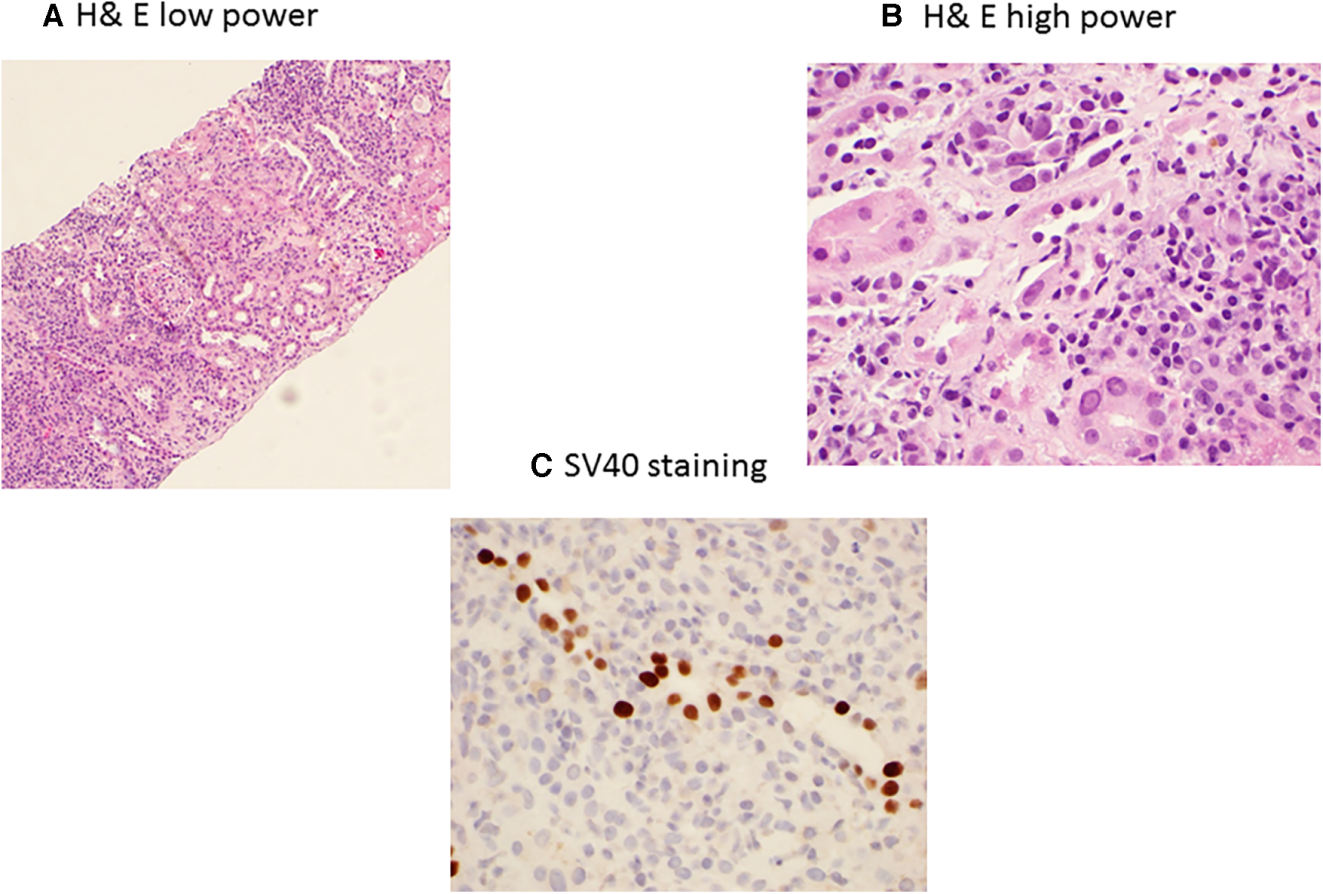
BK polyomavirus nephropathy staining with H&E low power (**A**) showing extensive interstitial inflammation, and high power (**B**) showing interstitial inflammation with mixed infiltrates of mononuclear and plasma cells, tubulitis, and nuclear enlargement, hyperchromasia and intranuclear inclusions of tubular epithelial cells; and immunohistochemical SV40 staining (**C**) showing positive nuclear SV40 staining in tubular epithelial cells.

BKPyVAN is the major complication of BKPyV replication. The incidence of BKPyVAN is highest in the first 2–6 months post transplant, with the majority of cases occurring within the first year of the transplant (54). A kidney allograft biopsy is necessary for the diagnosis of BKPyVAN (55). However, interpretation of the biopsy can be significantly complicated due to sampling variation and or concomitant rejection (56, 57). It is associated with characteristic histologic findings on kidney biopsy. The Banff Working Group in 2017 established the classification of BKPyVAN based on intrarenal polyomavirus replication/load levels (pvl) and Banff interstitial fibrosis (ci) scores from class I-3 (58). A tubule with intranuclear viral inclusion bodies and/or a positive immunohistochemical reaction for SV40 large T antigen in one or more cells per tubular cross-section is considered a positive tubule (58). BK staining using hematoxylin and eosin (H&E) in low power and high power along with SV40 staining is presented in Figure 2A–C.

**Figure 3:**
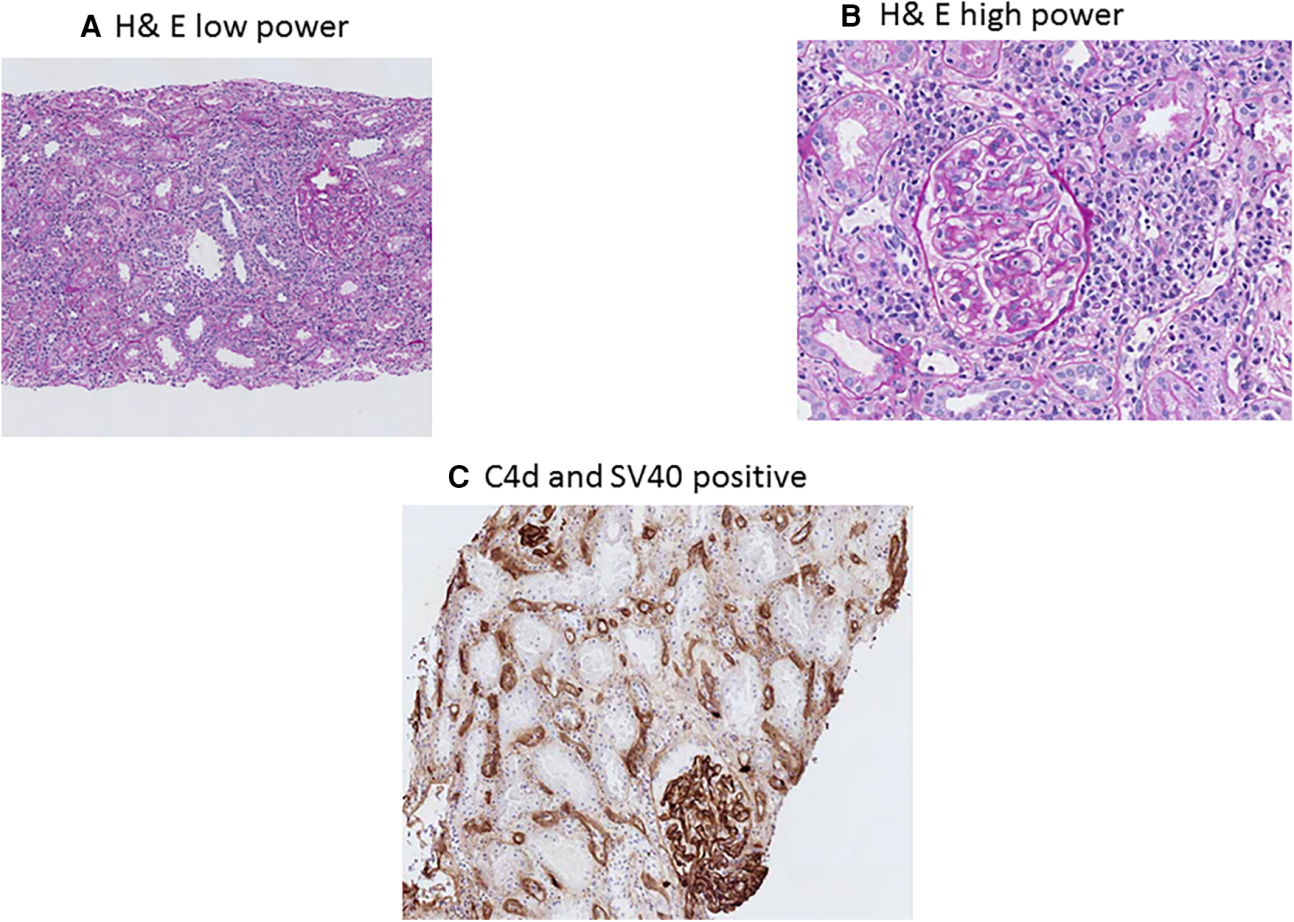
BK polyomavirus nephropathy and concurrent antibody rejection staining with PAS low-power (**A**) showing diffuse interstitial inflammation, and high-power (**B**) showing mononuclear interstitial inflammation, tubulitis, glomerulitis and peritubular capillaritis; immunohistochemical C4d staining (**C**) showing diffuse positive C4d staining in peritubular capillaries; and immunohostochemical SV40 staining.

**Figure 4:**
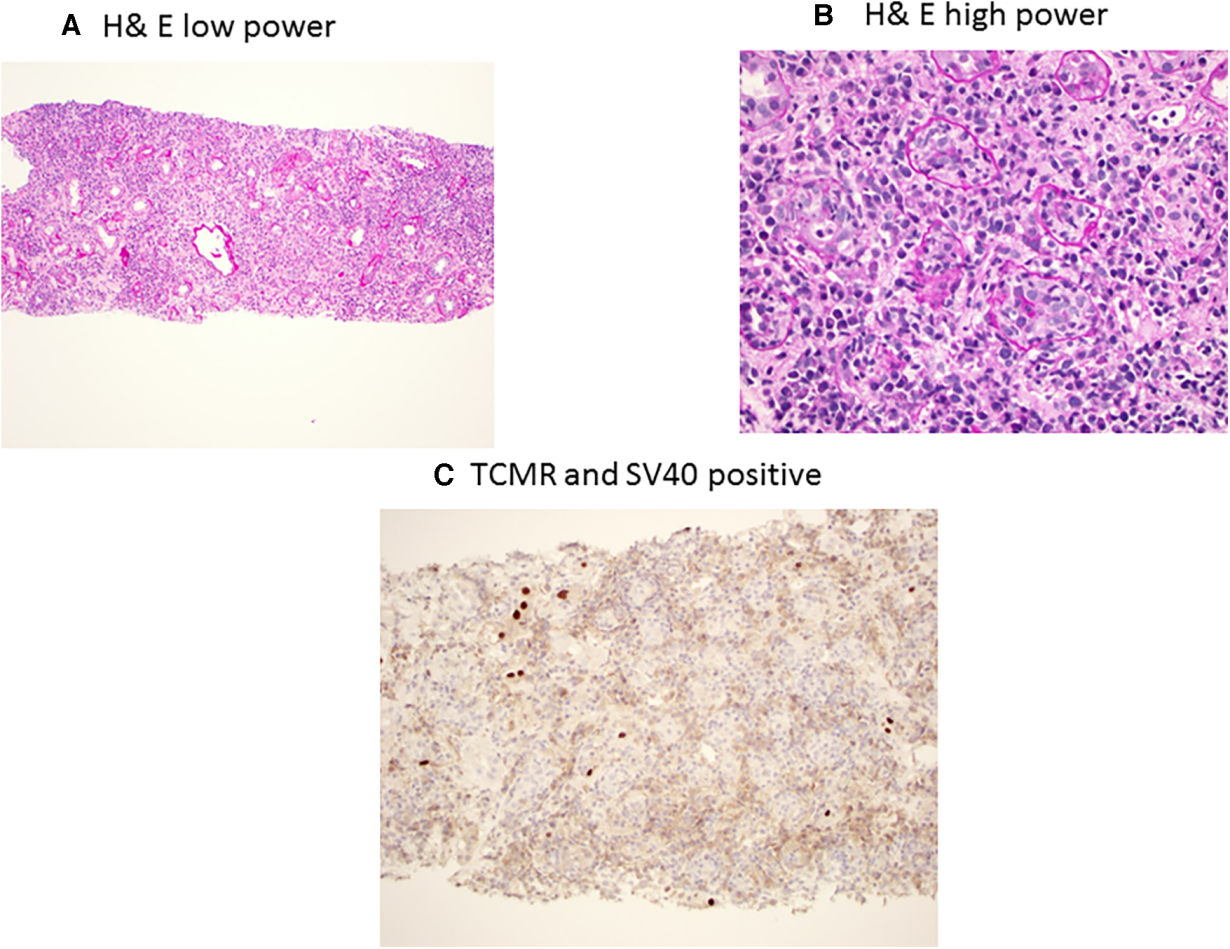
BK polyomavirus nephropathy and concurrent T-cell-mediated rejection staining with PAS low power (**A**) showing diffuse interstitial inflammation, and high power (**B**) showing interstitial inflammation with mixed mononuclear and plasma cell infiltrates with severe tubulitis; and immunohistochemical SV40 staining (**C**) showing focal tubules with scattered nuclear SV40 staining.

BKPyVAN and rejection are two extremes of immunosuppression management in transplantation, as BKPyVAN indicated an excess suppression of immunity, while rejection is due to hyperactive immune systems (59). However, in clinical practice, it is not uncommon to see simultaneous rejection and BKPyVAN. In one study, having early BKPyVAN and concurrent microvascular inflammation (a feature of antibody-mediated rejection (AMR)) and higher serum creatinine were associated with an increased risk of kidney allograft failure (60). AMR and BK staining using H&E in low power and high power along with C4d and SV40 staining is presented in Figure 3A–C. The differentiation between T-cell-mediated rejection (TCMR) and BKPyVAN is important but could be challenging. Some of the pathophysiological features could be similar and two entities could even co-exist (61). TCMR and BK staining using H&E in low power and high power along with SV40 staining is presented in Figure 4A–C. Rogers et al. compared 10 cases of BKPyVAN and 20 cases of TCMR and found similar CD20 staining in both groups (62). Similarly, Yamanaka et al. studied the immunohistochemical features of BKPyVAN and demonstrated that BKPyVAN primarily affects the collecting duct to the distal tubule (63). In TCMR, tubulitis affects mostly distal tubular segments in the cortex; proximal tubules are often spared, and collecting ducts in the medulla are hardly involved (64). Tubular epithelial cells are predominantly affected in BKPyVAN; however, it is not uncommon to find glomerular changes as well (65).

Infections and rejections are entangled, it is not uncommon that management of BKPyV viremia or nephropathy may trigger rejection, and treatment of rejection may increase the risk of severe BKPyV and BKPyVAN (66, 67). BKPyVAN and acute rejection are both nephrotoxic to the kidney and damage the kidney allograft. Mannon et al. found that patients with BKPyVAN demonstrated a significant elevation of transcripts for inflammatory cytokines and CD8+ T-cell cytotoxic molecules similar to TCMR but at higher levels of gene transcripts associated with graft fibrosis and of epithelial-mesenchymal damage (68). Similar to this finding, in one study of 96 cases of BKPyVAN and 256 cases of acute rejection, the difference in the rate of graft failure was similar in both groups, while at 3 years after diagnosis, kidney function was worse in the BKPyVAN group compared to the rejection group (69). Given all these findings, while rejection is a feared complication, BK polyomavirus nephropathy (BKPyVAN) is also an equally lethal complication.

### Management of concurrent BKPyVAN with acute rejection

There are conflicting data guiding the optimal management of concurrent BKPyVAN and acute rejection. Some suggest treating the acute rejection first followed by a subsequent reduction of immunosuppression after the patient achieved a clinical response with antirejection therapy (31), while others propose a further reduction of maintenance immunosuppression after the diagnosis of concurrent BKPyVAN and acute rejection (41, 70). In our clinical practice, we reduce or stop antimetabolites and add IVIG. It should be noted that there is no guideline about resuming previous immunosuppression once BKPyVAN and rejection are treated. In our practice, once serum BKPyV PCR is down-trending (usually serum BKPyV PCR <10,000 copies/ml), we reintroduce mycophenolic acid at a lower dose (approximately 25% of the original dose) and step up slowly with close monitoring of kidney function and BKPyV PCR.

### Persistent BKPyV viremia

Of all detectable BKPyV viremia, 50% occurs within the first 2 months and 95% within the first 2 years after transplant (71). There are variations in the study about the time of clearance of BKPyV viremia after a stepwise immunosuppression reduction. In one study, 78% of infected patients were still viremic 4 weeks after diagnosis and the initiation of immunosuppression reduction, and 61.5% of viremic at 3 months (52). For most individuals, persistent infections at low levels are clinically silent and not associated with adverse outcomes (66, 72). However, persistent high BKPyV viremia was associated with BKPyVAN and graft dysfunction (66). BKPyV can also play a direct persistent causal role in bladder carcinoma and other genitourinary cancers. Persistent BKPyV may warrant cystoscopy and evaluation for bladder cancer (73).

#### Other complications

Although rare in kidney transplant recipients, BKPyV is known to cause ureteric stenosis and hemorrhagic cystitis (74). There is a known link between BKPyV and the development of genitourinary cancers mainly in the animal models (75, 76).

### Retransplant after graft failure due to BKPyVAN

Graft failure after BKPyVAN is a common complication; in the USA it is estimated that approximately 300 kidney grafts fail due to BKPyVAN every year (77). It is not uncommon for these recipients to seek another kidney transplant. Current guidelines also support retransplant among recipients who had a previous graft failure due to BKPyVAN (31). In a current study among second kidney transplants between 2005 and 2016, with 13,601 recipients, the authors compared first kidney failure due to BKPyVAN vs. other causes, with a median follow-up of 4.7 years, and found similar outcomes in terms of death-censored graft survival, acute rejection, or patient survival (78). The absence of BKPyV replication should be confirmed before retransplantation (71). Most of the centers wait for the resolution of BKPyV viremia. However, successful pre-emptive, living, related kidney transplants during active BKPyVAN with viremia have been reported in two recipients with simultaneous graft nephrectomy (79). Even a case of successful repeat kidney transplant in a patient with high-grade BKPyV viremia and fulminant hepatic failure without concomitant allograft nephrectomy has been reported (80). Some centers consider failed allograft and/or native nephrectomy before considering retransplant. However, this is not recommended, given the lack of evidence-based guidelines to substantiate this practice (50).

## Discussion

In this article, we summarized various pitfalls of BKPyV infection in kidney transplant recipients starting from the recognition of the risk factors, screening methods, treatment, graft failure, and retransplant, despite being such a common infection that infects almost one-third of kidney recipients. However, recently, among the transplant community, this infection has gotten more attention. Most of the centers have heightened surveillance protocols leading to early detection. There are also multiple clinical trials in the pipeline for the treatment of BKPyV. There are multiple unknowns about the course of this infection. It would have been better if we were able to assess the risk of progression to a severe form of BKPyV, i.e., BKPyVAN among those with early detection of BKPyV viremia. As mentioned earlier, even after protocolized immunosuppression reduction, 28% of recipients developed a severe form of BKPyV and 19% developed rejection or *de novo* Donor specific antibody (DSA) (42). In addition, although the direct burden of graft failure attributed to BKPyV may not sound that high, there could be indirect consequences related to BKPyV replication. For example, among recipients with BKPyV viremia, detected on routine screening, and immunosuppression reduction, if they develop rejection and graft failure, the cause of the graft failure will be attributed to the rejection rather than BKPyV, which leads to rejection. Further, the costs associated with the management of BKPyV and other indirect consequences, including psychological stress recipients have to deal with, are understudied.

In conclusion, BKPyV infection is associated with significant morbidity and mortality after kidney transplantation. BKPyV infection is, in general, asymptomatic and currently only diagnosed with routine screening. Despite having 50 years of experience, the management of BKPyV infection remains limited and controversial. In the absence of effective antiviral medications and with the emergence of potent immunosuppressive medications to treat and prevent rejection, we may win the battle against rejection, but lose the war of graft failure due to BKPyV. With the emergence of VST therapy, we hope to see some positive outcomes in the near future.

## Data Availability

The original contributions presented in the study are included in the article/Supplementary Material, further inquiries can be directed to the corresponding author.

## References

[1] Hirsch HH Randhawa P, A.S.T.I.D.C.o. Practice. BK Polyomavirus in solid organ transplantation. Am J Transplant. (2013) 13(Suppl 4):179–88. 10.1111/ajt.12110 23465010

[2] Gardner S Field A Coleman D Hulme B. New human papovavirus (BK) isolated from urine after renal transplantation. Lancet. (1971) 297(7712):1253–7. 10.1016/S0140-6736(71)91776-4 4104714

[3] Myint TM Chong CHY Wyld M Nankivell B Kable K Wong G. Polyoma BK virus in kidney transplant recipients: screening, monitoring, and management. Transplantation. (2022) 106(1):e76–89. 10.1097/tp.0000000000003801 33908382

[4] Popik W Khatua AK Fabre NF Hildreth JEK Alcendor DJ. BK Virus replication in the glomerular vascular unit: implications for BK virus associated nephropathy. Viruses. (2019) 11(7):583. 10.3390/v11070583 31252545 PMC6669441

[5] Sawinski D Goral S. BK virus infection: an update on diagnosis and treatment. Nephrol Dial Transplant. (2014) 30(2):209–17. 10.1093/ndt/gfu023 24574543

[6] Hirsch HH Vincenti F Friman S Tuncer M Citterio F Wiecek A Polyomavirus BK replication in de novo kidney transplant patients receiving tacrolimus or cyclosporine: a prospective, randomized, multicenter study. Am J Transplant. (2013) 13(1):136–45. 10.1111/j.1600-6143.2012.04320.x 23137180 PMC3563214

[7] Cohen-Bucay A Ramirez-Andrade SE Gordon CE Francis JM Chitalia VC. Advances in BK virus complications in organ transplantation and beyond. Kidney Med. (2020) 2(6):771–86. 10.1016/j.xkme.2020.06.015 33319201 PMC7729234

[8] Avcı B Baskın E Gülleroğlu K Ecevit Z Ayvazoğlu Soy E Moray G BK polyomavirus infection and risk factors in pediatric patients undergoing kidney transplant. Exp Clin Transplant. (2022) 20(Suppl 3):105–11. 10.6002/ect.PediatricSymp2022.O34 35570612

[9] Luo C Bueno M Kant J Martinson J Randhawa P. Genotyping schemes for polyomavirus BK, using gene-specific phylogenetic trees and single nucleotide polymorphism analysis. J Virol. (2009) 83(5):2285–97. 10.1128/JVI.02180-08 19109389 PMC2643714

[10] Zhong S Randhawa PS Ikegaya H Chen Q Zheng HY Suzuki M Distribution patterns of BK polyomavirus (BKV) subtypes and subgroups in American, European and Asian populations suggest co-migration of BKV and the human race. J Gen Virol. (2009) 90(Pt 1):144–52. 10.1099/vir.0.83611-0 19088283

[11] Gossai A Waterboer T Nelson HH Michel A Willhauck-Fleckenstein M Farzan SF Seroepidemiology of human polyomaviruses in a US population. Am J Epidemiol. (2016) 183(1):61–9. 10.1093/aje/kwv155 26667254 PMC5006224

[12] Pastrana DV Ray U Magaldi TG Schowalter RM Cuburu N Buck CB. BK Polyomavirus genotypes represent distinct serotypes with distinct entry tropism. J Virol. (2013) 87(18):10105–13. 10.1128/JVI.01189-13 23843634 PMC3754014

[13] Stolt A Sasnauskas K Koskela P Lehtinen M Dillner J. Seroepidemiology of the human polyomaviruses. J Gen Virol. (2003) 84(Pt 6):1499–504. 10.1099/vir.0.18842-0 12771419

[14] Yogo Y Sugimoto C Zhong S Homma Y. Evolution of the BK polyomavirus: epidemiological, anthropological and clinical implications. Rev Med Virol. (2009) 19(4):185–99. 10.1002/rmv.613 19530118

[15] Zheng HY Nishimoto Y Chen Q Hasegawa M Zhong S Ikegaya H Relationships between BK virus lineages and human populations. Microbes Infect. (2007) 9(2):204–13. 10.1016/j.micinf.2006.11.008 17208484

[16] Solis M Velay A Porcher R Domingo-Calap P Soulier E Joly M Neutralizing antibody-mediated response and risk of BK virus-associated nephropathy. J Am Soc Nephrol. (2018) 29(1):326–34. 10.1681/ASN.2017050532 29042457 PMC5748919

[17] Borni-Duval C Caillard S Olagne J Perrin P Braun-Parvez L Heibel F Risk factors for BK virus infection in the era of therapeutic drug monitoring. Transplantation. (2013) 95(12):1498–505. 10.1097/TP.0b013e3182921995 23778568

[18] Radtke J Dietze N Fischer L Achilles EG Li J Scheidat S Incidence of BK polyomavirus infection after kidney transplantation is independent of type of immunosuppressive therapy. Transpl Infect Dis. (2016) 18(6):850–5. 10.1111/tid.12611 27639176

[19] Haririan A Klassen DK. BK Virus infection after nonrenal transplantation. Graft-Georgetown-. (2002) 5:S58–64. 10.1177/1522162802238458

[20] Breyer I Dodin B Djamali A Jorgenson MR Garg N Aziz F Risk factors and outcomes of BK viremia among deceased donor kidney transplant recipients based on donor characteristics. Transpl Infect Dis. (2022) 24(1):e13768. 10.1111/tid.13768 34825437

[21] Drachenberg CB Papadimitriou JC Mann D Hirsch HH Wali R Ramos E. Negative impact of human leukocyte antigen matching in the outcome of polyomavirus nephropathy. Transplantation. (2005) 80(2):276–8. 10.1097/01.tp.0000165096.01034.15 16041275

[22] Parajuli S Muth BL Turk JA Astor BC Mohammed M Mandelbrot DA In kidney transplant recipients with a positive virtual crossmatch, high PRA was associated with lower incidence of viral infections. Transplantation. (2016) 100(3):655–61. 10.1097/tp.0000000000001061 26760571

[23] Alalawi F El Kossi M Jenkins J Halawa A. BK Virus infection in adult renal transplant recipients; risk factors and their impact on allograft survival. Trends Transplant. (2020) 13:2020. 10.15761/TiT.1000278

[24] Bohl DL Storch GA Ryschkewitsch C Gaudreault-Keener M Schnitzler MA Major EO Donor origin of BK virus in renal transplantation and role of HLA C7 in susceptibility to sustained BK viremia. Am J Transplant. (2005) 5(9):2213–21. 10.1111/j.1600-6143.2005.01000.x 16095500

[25] Srivastava A Bodnar J Osman F Jorgenson MR Astor BC Mandelbrot DA Serum albumin level before kidney transplant predicts post-transplant BK and possibly cytomegalovirus infection. Kidney Int Rep. (2020) 5(12):2228–37. 10.1016/j.ekir.2020.09.012 33305116 PMC7710825

[26] Alshaikh EA Astor BC Muth B Jorgenson M Swanson K Garg N Delayed graft function among kidney transplant recipients is associated with an increased risk of urinary tract infection and BK viremia. Transplant Direct. (2023) 9(9):e1526. 10.1097/txd.0000000000001526 37654682 PMC10466499

[27] Bressollette-Bodin C Coste-Burel M Hourmant M Sebille V Andre-Garnier E Imbert-Marcille BM. A prospective longitudinal study of BK virus infection in 104 renal transplant recipients. Am J Transplant. (2005) 5(8):1926–33. 10.1111/j.1600-6143.2005.00934.x 15996241

[28] Thangaraju S Gill J Wright A Dong J Rose C Gill J. Risk factors for BK polyoma virus treatment and association of treatment with kidney transplant failure: insights from a paired kidney analysis. Transplantation. (2016) 100(4):854–61. 10.1097/tp.0000000000000890 27003098

[29] Atencio IA Shadan FF Zhou XJ Vaziri ND Villarreal LP. Adult mouse kidneys become permissive to acute polyomavirus infection and reactivate persistent infections in response to cellular damage and regeneration. J Virol. (1993) 67(3):1424–32. 10.1128/jvi.67.3.1424-1432.1993 8382304 PMC237512

[30] Hall IE Reese PP Mansour SG Mohan S Jia Y Thiessen-Philbrook HR Deceased-donor acute kidney injury and BK polyomavirus in kidney transplant recipients. Clin J Am Soc Nephrol. (2021) 16(5):765–75. 10.2215/cjn.18101120 33692117 PMC8259491

[31] Hirsch HH Randhawa PS. BK polyomavirus in solid organ transplantation-guidelines from the American society of transplantation infectious diseases community of practice. Clin Transplant. (2019) 33(9):e13528. 10.1111/ctr.13528 30859620

[32] Safa K Heher E Gilligan H Williams W Jr. Tolkoff-Rubin N Wojciechowski D. BK virus after kidney transplantation: a review of screening and treatment strategies and a summary of the massachusetts general hospital experience. Clin Transpl. (2015) 31:257–63. PMID: 28514587. 28514587

[33] Baker RJ Mark PB Patel RK Stevens KK Palmer N. Renal association clinical practice guideline in post-operative care in the kidney transplant recipient. BMC Nephrol. (2017) 18(1):174. 10.1186/s12882-017-0553-2 28571571 PMC5455080

[34] Nickeleit V Klimkait T Binet IF Dalquen P Del Zenero V Thiel G Testing for polyomavirus type BK DNA in plasma to identify renal-allograft recipients with viral nephropathy. N Engl J Med. (2000) 342(18):1309–15. 10.1056/nejm200005043421802 10793163

[35] Haller J Diebold M Leuzinger K Wehmeier C Handschin J Amico P Urine CXCL10 to assess BK polyomavirus replication after kidney transplantation. Transplantation. (2023) 107(12):2568–74. 10.1097/tp.0000000000004712 37408094 PMC10664791

[36] Hertz-Tang AL Astor BC Mandelbrot DA Mohamed MA Djamali A Parajuli S. BK viremia is not associated with adverse outcomes in the absence of BK nephropathy. Clin Transplant. (2018) 32(7):e13283. 10.1111/ctr.13283 29774593

[37] Furmaga J Kowalczyk M Zapolski T Furmaga O Krakowski L Rudzki G BK polyomavirus-biology, genomic variation and diagnosis. Viruses. (2021) 13(8):1502. 10.3390/v13081502 34452367 PMC8402805

[38] Morel V Martin E François C Helle F Faucher J Mourez T A simple and reliable strategy for BK virus subtyping and subgrouping. J Clin Microbiol. (2017) 55(4):1177–85. 10.1128/jcm.01180-16 28151406 PMC5377845

[39] Coleman DV Mackenzie EF Gardner SD Poulding JM Amer B Russell WJ. Human polyomavirus (BK) infection and ureteric stenosis in renal allograft recipients. J Clin Pathol. (1978) 31(4):338–47. 10.1136/jcp.31.4.338 205555 PMC1145271

[40] Gonzalez S Escobar-Serna DP Suarez O Benavides X Escobar-Serna JF Lozano E. BK virus nephropathy in kidney transplantation: an approach proposal and update on risk factors, diagnosis, and treatment. Transplant Proc. (2015) 47(6):1777–85. 10.1016/j.transproceed.2015.05.010 26293050

[41] Schaub S Hirsch HH Dickenmann M Steiger J Mihatsch MJ Hopfer H Reducing immunosuppression preserves allograft function in presumptive and definitive polyomavirus-associated nephropathy. Am J Transplant. (2010) 10(12):2615–23. 10.1111/j.1600-6143.2010.03310.x 21114642

[42] Kharel A Djamali A Jorgenson MR Alzoubi B Swanson KJ Garg N Risk factors for progression from low level BK dnaemia to unfavorable outcomes after BK management via immunosuppressive reduction. Transpl Infect Dis. (2021) 23(3):e13561. 10.1111/tid.13561 33400361

[43] Parajuli S Jorgenson M Meyers RO Djamali A Galipeau J. Role of virus-specific T cell therapy for cytomegalovirus and BK infections in kidney transplant recipients. Kidney360. (2021) 2(5):905–15. 10.34067/KID.0001572021 35373059 PMC8791350

[44] Lamarche C Orio J Georges-Tobar V Pincez T Goupil M Dahmani A Clinical-scale rapid autologous BK virus-specific T cell line generation from kidney transplant recipients with active viremia for adoptive immunotherapy. Transplantation. (2017) 101(11):2713–21. 10.1097/tp.0000000000001698 28230645

[45] Peggs KS Verfuerth S Pizzey A Khan N Guiver M Moss PA Adoptive cellular therapy for early cytomegalovirus infection after allogeneic stem-cell transplantation with virus-specific T-cell lines. Lancet. (2003) 362(9393):1375–7. 10.1016/s0140-6736(03)14634-x 14585640

[46] Zhong S Zheng HY Suzuki M Chen Q Ikegaya H Aoki N Age-related urinary excretion of BK polyomavirus by nonimmunocompromised individuals. J Clin Microbiol. (2007) 45(1):193–8. 10.1128/jcm.01645-06 17093017 PMC1828952

[47] Lee S Lee KW Kim SJ Park JB. Clinical characteristic and outcomes of BK virus infection in kidney transplant recipients managed using a systematic surveillance and treatment strategy. Transplant Proc. (2020) 52(6):1749–56. 10.1016/j.transproceed.2020.01.158 32402452

[48] Elfadawy N Yamada M Sarabu N. Management of BK polyomavirus infection in kidney and kidney-pancreas transplant recipients: a review article. Infect Dis Clin North Am. (2018) 32(3):599–613. 10.1016/j.idc.2018.04.009 30146025

[49] Drachenberg CB Beskow CO Cangro CB Bourquin PM Simsir A Fink J Human polyoma virus in renal allograft biopsies: morphological findings and correlation with urine cytology. Hum Pathol. (1999) 30(8):970–7. 10.1016/s0046-8177(99)90252-6 10452511

[50] Kant S Dasgupta A Bagnasco S Brennan DC. BK virus nephropathy in kidney transplantation: a state-of-the-art review. Viruses. (2022) 14(8):1616. 10.3390/v14081616 35893681 PMC9330039

[51] Madden K Janitell C Sower D Yang S. Prediction of BK viremia by urine viral load in renal transplant patients: an analysis of BK viral load results in paired urine and plasma samples. Transpl Infect Dis. (2018) 20(5):e12952. 10.1111/tid.12952 29896858

[52] Sawinski D Forde KA Trofe-Clark J Patel P Olivera B Goral S Persistent BK viremia does not increase intermediate-term graft loss but is associated with de novo donor-specific antibodies. J Am Soc Nephrol. (2015) 26(4):966–75. 10.1681/asn.2014010119 25255921 PMC4378106

[53] Sawinski D Trofe-Clark J. BKV viremia and development of De Novo DSA in renal transplant recipients. Clin Transpl. (2015) 31:249–56. PMID: 28514586. 28514586

[54] Imlay H Whitaker K Fisher CE Limaye AP. Clinical characteristics and outcomes of late-onset BK virus nephropathy in kidney and kidney-pancreas transplant recipients. Transpl Infect Dis. (2018) 20(4):e12928. 10.1111/tid.12928 29809315

[55] Drachenberg CB Papadimitriou JC Chaudhry MR Ugarte R Mavanur M Thomas B Histological evolution of BK virus–associated nephropathy: importance of integrating clinical and pathological findings. Am J Transplant. (2017) 17(8):2078–91. 10.1111/ajt.14314 28422412

[56] McGregor SM Chon WJ Kim L Chang A Meehan SM. Clinical and pathological features of kidney transplant patients with concurrent polyomavirus nephropathy and rejection-associated endarteritis. World J Transplant. (2015) 5(4):292. 10.5500/wjt.v5.i4.292 26722657 PMC4689940

[57] Drachenberg CB Papadimitriou JC Hirsch HH Wali R Crowder C Nogueira J Histological patterns of polyomavirus nephropathy: correlation with graft outcome and viral load. Am J Transplant. (2004) 4(12):2082–92. 10.1046/j.1600-6143.2004.00603.x 15575913

[58] Nickeleit V Singh HK Randhawa P Drachenberg CB Bhatnagar R Bracamonte E The Banff working group classification of definitive polyomavirus nephropathy: morphologic definitions and clinical correlations. J Am Soc Nephrol. (2018) 29(2):680–93. 10.1681/asn.2017050477 29279304 PMC5791071

[59] Shen CL Wu BS Lien TJ Yang AH Yang CY. BK Polyomavirus nephropathy in kidney transplantation: balancing rejection and infection. Viruses. (2021) 13(3):487. 10.3390/v13030487 33809472 PMC7998398

[60] Mohamed M Parajuli S Muth B Astor BC Panzer SE Mandelbrot D In kidney transplant recipients with BK polyomavirus infection, early BK nephropathy, microvascular inflammation, and serum creatinine are risk factors for graft loss. Transpl Infect Dis. (2016) 18(3):361–71. 10.1111/tid.12530 26998753

[61] Jamboti JS. BK virus nephropathy in renal transplant recipients. Nephrology. (2016) 21(8):647–54. 10.1111/nep.12728 26780694

[62] Rogers NM Russ GR Cooper J Coates PT. Immunophenotyping of interstitial infiltrate does not distinguish between BK virus nephropathy and acute cellular rejection. Nephrology (Carlton). (2009) 14(1):118–22. 10.1111/j.1440-1797.2008.01050.x 19143944

[63] Yamanaka K Oka K Nakazawa S Hirai T Kishikawa H Nishimura K Immunohistochemical features of BK virus nephropathy in renal transplant recipients. Clin Transplant. (2012) 26(Suppl 24):20–4. 10.1111/j.1399-0012.2012.01636.x 22747471

[64] Iványi B Hansen HE Olsen S. Segmental localization and quantitative characteristics of tubulitis in kidney biopsies from patients undergoing acute rejection. Transplantation. (1993) 56(3):581–5. 10.1097/00007890-199309000-00017 8212153

[65] Celik B Randhawa PS. Glomerular changes in BK virus nephropathy. Hum Pathol. (2004) 35(3):367–70. 10.1016/j.humpath.2003.09.009 15017594

[66] Elfadawy N Flechner SM Schold JD Srinivas TR Poggio E Fatica R Transient versus persistent BK viremia and long-term outcomes after kidney and kidney-pancreas transplantation. Clin J Am Soc Nephrol. (2014) 9(3):553–61. 10.2215/cjn.08420813 24408118 PMC3944774

[67] Gately R Milanzi E Lim W Teixeira-Pinto A Clayton P Isbel N Incidence, risk factors, and outcomes of kidney transplant recipients with BK polyomavirus-associated nephropathy. Kidney Int Rep. (2023) 8(3):531–43. 10.1016/j.ekir.2022.12.020 36938086 PMC10014440

[68] Mannon RB Hoffmann SC Kampen RL Cheng OC Kleiner DE Ryschkewitsch C Molecular evaluation of BK polyomavirus nephropathy. Am J Transplant. (2005) 5(12):2883–93. 10.1111/j.1600-6143.2005.01096.x 16303001

[69] Parajuli S Astor BC Kaufman D Muth B Mohamed M Garg N Which is more nephrotoxic for kidney transplants: bK nephropathy or rejection? Clin Transplant. (2018) 32(4):e13216. 10.1111/ctr.13216 29394515

[70] Brennan DC Agha I Bohl DL Schnitzler MA Hardinger KL Lockwood M Incidence of BK with tacrolimus versus cyclosporine and impact of preemptive immunosuppression reduction. Am J Transplant. (2005) 5(3):582–94. 10.1111/j.1600-6143.2005.00742.x 15707414

[71] Hirsch HH Brennan DC Drachenberg CB Ginevri F Gordon J Limaye AP Polyomavirus-associated nephropathy in renal transplantation: interdisciplinary analyses and recommendations. Transplantation. (2005) 79(10):1277–86. 10.1097/01.tp.0000156165.83160.09 15912088

[72] Chakrabarti U Chaturvedy M Bajpai NK Goswami J Garsa RK Jhorawat R. BK Virus infection and its management in renal transplantation: an update. OBM Transplantation. (2023) 7(3):1–23. 10.21926/obm.transplant.2303192

[73] Prom A Jorgenson M Alagusundaramoorthy S Parajuli S. Persistent BK polyomavirus-DNAemia may warrant cystoscopy to rule out urologic carcinoma: a case report and review of the literature. Transpl Infect Dis. (2020) 22(5):e13316. 10.1111/tid.13316 32386093

[74] Rajpoot DK Gomez A Tsang W Shanberg A. Ureteric and urethral stenosis: a complication of BK virus infection in a pediatric renal transplant patient. Pediatr Transplant. (2007) 11(4):433–5. 10.1111/j.1399-3046.2006.00673.x 17493226

[75] Abend JR Jiang M Imperiale MJ. BK virus and human cancer: innocent until proven guilty. Semin Cancer Biol. (2009) 19(4):252–60. 10.1016/j.semcancer.2009.02.004 19505653 PMC2694748

[76] Geetha D Tong BC Racusen L Markowitz JS Westra WH. Bladder carcinoma in a transplant recipient: evidence to implicate the BK human polyomavirus as a causal transforming agent. Transplantation. (2002) 73(12):1933–6. 10.1097/00007890-200206270-00015 12131691

[77] Geetha D Sozio SM Ghanta M Josephson M Shapiro R Dadhania D Results of repeat renal transplantation after graft loss from BK virus nephropathy. Transplantation. (2011) 92(7):781–6. 10.1097/TP.0b013e31822d08c1 21836535

[78] Leeaphorn N Thongprayoon C Chon WJ Cummings LS Mao MA Cheungpasitporn W. Outcomes of kidney retransplantation after graft loss as a result of BK virus nephropathy in the era of newer immunosuppressant agents. Am J Transplant. (2020) 20(5):1334–40. 10.1111/ajt.15723 31765056

[79] Womer KL Meier-Kriesche HU Patton PR Dibadj K Bucci CM Foley D Preemptive retransplantation for BK virus nephropathy: successful outcome despite active viremia. Am J Transplant. (2006) 6(1):209–13. 10.1111/j.1600-6143.2005.01137.x 16433777

[80] Huang J Danovitch G Pham PT Bunnapradist S Huang E. Kidney retransplantation for BK virus nephropathy with active viremia without allograft nephrectomy. J Nephrol. (2015) 28(6):773–7. 10.1007/s40620-015-0200-6 25910469

